# Microbial profile comparisons of saliva, pooled and site-specific subgingival samples in periodontitis patients

**DOI:** 10.1371/journal.pone.0182992

**Published:** 2017-08-11

**Authors:** Daniel Belstrøm, Maria Lynn Sembler-Møller, Maria Anastasia Grande, Nikolai Kirkby, Sean Liam Cotton, Bruce J. Paster, Palle Holmstrup

**Affiliations:** 1 Section for Periodontology, Microbiology, and Community Dentistry, Department of Odontology, Faculty of Health Sciences, University of Copenhagen, Copenhagen, Denmark; 2 Section for Oral Medicine, Department of Odontology, Faculty of Health Sciences, University of Copenhagen, Copenhagen, Denmark; 3 Department of Medical Microbiology, Copenhagen University Hospital, Copenhagen, Denmark; 4 The Forsyth Institute, Cambridge, MA, United States of America; 5 Department of Oral Medicine, Infection & Immunity, Harvard School of Dental Medicine, Boston, MA, United States of America; Medical University of South Carolina, UNITED STATES

## Abstract

**Objectives:**

The purpose of this study was to compare microbial profiles of saliva, pooled and site-specific subgingival samples in patients with periodontitis. We tested the hypotheses that saliva can be an alternative to pooled subgingival samples, when screening for presence of periopathogens.

**Design:**

Site specific subgingival plaque samples (n = 54), pooled subgingival plaque samples (n = 18) and stimulated saliva samples (n = 18) were collected from 18 patients with generalized chronic periodontitis. Subgingival and salivary microbiotas were characterized by means of HOMI*NGS* (Human Oral Microbe Identification using Next Generation Sequencing) and microbial community profiles were compared using Spearman rank correlation coefficient.

**Results:**

Pronounced intraindividual differences were recorded in site-specific microbial profiles, and site-specific information was in general not reflected by pooled subgingival samples. Presence of *Porphyromonas gingivalis*, *Treponema denticola*, *Prevotella intermedia*, *Filifactor alocis*, *Tannerella forsythia* and *Parvimona micra* in site-specific subgingival samples were detected in saliva with an AUC of 0.79 (sensitivity: 0.61, specificity: 0.94), compared to an AUC of 0.76 (sensitivity: 0.56, specificity: 0.94) in pooled subgingival samples.

**Conclusions:**

Site-specific presence of periodontal pathogens was detected with comparable accuracy in stimulated saliva samples and pooled subgingival plaque samples. Consequently, saliva may be a reasonable surrogate for pooled subgingival samples when screening for presence of periopathogens. Future large-scale studies are needed to confirm findings from this study.

## Introduction

The oral cavity is harbored by a complex and diverse microbiota, which comprises more than 700 different predominant bacterial species [1]. The oral microbiota shows a high degree of site-specific characteristics [2], which is shaped by ecological conditions and perturbations in different oral locations [3;4]. A symbiotic relationship between the resident oral microbiota and the host is essential for oral homeostasis, while alteration of the subgingival microbiota is critically involved in development of periodontitis [5].

Subgingival colonization by specific species, including the red complex bacterial species, i.e., *Porphyromonas gingivalis*, *Tannerella forsythia* and *Treponema denticola* has been reported to be strongly associated with progression of periodontitis as determined by using DNA-DNA checkerboard analysis [6]. The development of contemporary molecular methods has expanded the list of potential periodontal pathogens, including *Parvimonas micra* and *Filifactor alocis* [7–10]. Analysis of subgingival plaque is considered the gold standard when studying periodontitis-associated microbial profiles. Ideally, analysis of single-site should be performed, but for practical and economic reasons, pooled subgingival plaque samples have often been employed [11]. However, it is well-known that detailed site-specific information might get lost when pooled samples are employed for microbial analysis [12].

Collection of subgingival plaque samples can be a tedious procedure, which can only be performed by trained dental professionals. On the other hand, saliva is easily obtained, even at home by the patients themselves [13;14]. Thus, saliva has been suggested an alternative to local microbial samples for analysis of periodontitis-associated biomarkers [15]. Salivary presence and relative abundance of red complex periodontal pathogens associates with periodontitis [16;17], and several studies have reported a positive correlation between presence of specific periopathogens in pooled subgingival samples and saliva samples [18–21]. However, to the best of our knowledge a direct comparison on the efficacy of using saliva samples as an alternative to pooled subgingival samples for screening of site-specific presence of periopathogens has not been performed.

Thus, the purpose of this study was two-fold. 1: to characterize intra-individual variations in site-specific subgingival samples. 2: to compare the efficacy of using saliva samples and pooled subgingival samples for screening of site-specific presence of specific periopathogens. We tested the hypotheses that saliva can be an alternative to pooled subgingival samples, when screening for presence of periopathogens.

## Materials and methods

### Study design

Using data from a previous study, a sample size of n = 18 was estimated based on a power calculation with a difference in mean value of Shannon index of 0.1 as primary outcome (α = 0.05, β = 0.20, E = 0.10, S(Δ) = 0.15) [22]. Therefore 18 patients (11 male and 7 females, mean age: 54 yrs.) attending Copenhagen University School of Dentistry for periodontitis treatment were included in this study (Table 1). Patients were screened for eligibility based on full-mouth recordings of probing pocket depth (PPD), clinical attachment level (CAL) and bleeding on probing (BOP) at six sites on each tooth. Inclusion criteria were as follows: minimum 4 teeth with moderate to severe periodontitis as defined by the American Academy of Periodontology [23]. Exclusion criteria were as follows: use of antibiotics within the last 3 months, systemic diseases, and/or use of medication with known effect on periodontitis. All participants signed informed consents prior to participation. The study was approved by the regional ethical committee of the capital region of Denmark (H-16016368) and reported to the Danish Data Authority (SUND-2016-58).

**Table 1:** Demographic and periodontal clinical features of the sample population.

	Background data
**Age (mean, range)**	54 (38–75)
**Gender (M/F)**	11/7
**Smoking status (Y/N)**	10/8
PPD (mean, range)*	7 (5–10)
CAL (mean, range)*	8 (5–14)
Number of teeth with PPD≥5 mm + BOP**	14 (7–27)
% teeth with PPD≥5 mm + BOP**	53 (25–96)

* PPD and CAL measured in mm and expressed as mean and range from sites sampled (n = 3) from each subject.

** Teeth with PPD≥5 mm+BOP expressed as mean and range based on full mouth registration.

### Sample collection

All samples were collected between 8AM and 2 PM and before any dental treatment had been performed. First, a stimulated saliva sample was collected as previously described [24]. Second, subgingival plaque samples were collected from the three sites with the deepest periodontal pockets by use of sterile paper points according to a modified protocol from [8]. Paper points were placed in sterile saline immediately after collection, and all samples were stored at -80 C until further analysis. Microbial analysis was performed on pooled subgingival samples (combining one paper point from each site in the same tube) and site-specific samples (separate analysis of one paper point from each site).

### HOMINGS

DNA was extracted in accordance with the manufacturer’s guidelines (protocol: Pathogen_Universal_200, Roche, Mannheim, Germany).

The laboratory procedures of HOMI*NGS* have been presented in detail [22;25]. Initially, DNA concentrations were measured by a NanoDrop 8000 spectrophotometer (Thermo Scientific) followed by PCR-based amplification of bacterial DNA (10–50 ng) using forward (341F) and reverse (806R) primers targeting the V3-V4 region of the genes coding for 16S rRNA. Secondly, amplicons were purified using AMPure beads and libraries (100 ng) were pooled, gel-purified, and quantified using qPCR. Thirdly, samples were processed by next-generation sequencing (MiSeq, Illumina), according to a protocol modified from [26]. Finally, after removal of bad reads and chimeric sequences > 3.5 M sequences were included in further analysis.

### Statistical analysis

16S rDNA reads were BLASTed against a customized BLAST program (ProbeSeq for HOMI*NGS*) developed at the Forsyth Institute, Cambridge, USA [27] and assigned taxonomically at genus and species level, respectively. Samples were characterized based on relative abundance of identified taxa and α-diversity (Shannon index), and microbial community profiles were compared between samples using Spearman rank correlation coefficient. Data from site-specific samples from each patient was averaged and compared to data from pooled subgingival samples, to address if a pooled subgingival sample legitimately provides combined information on site-specific microbial profiles. To test the efficacy of using saliva and pooled subgingival samples in screening for periopathogens, presence of 6 bacterial species (*P*. *gingivalis*, *T*. *denticola*, *Prevotella intermedia*, *F*. *alocis*, *T*. *forsythia* and *P*. *micra*) in saliva and pooled subgingival samples were compared to site-specific data on the individual level. AUC, sensitivity, and specificity were computed for each screening method GraphPad prism 7 (San Diego, California, USA) and MeV 4_8_1 [28] was used as statistical software.

## Results

### General information

A total of 3,552,616 unique sequences were retrieved from 90 microbial samples (saliva: n = 18, pooled subgingival samples: n = 18, site-specific subgingival samples: n = 54) using high throughput next-generation sequencing. The mean number of DNA reads per sample was 39,474 (range: 14,414–107,796) with a significantly higher number of reads in single-site (40,783) and pooled (43,952) subgingival samples compared to stimulated saliva samples (31,067) (p<0.0001).

A total number of 487 different bacterial species were identified with a mean of 142 (range: 48–220) bacterial species per sample. A complete list of all identified species is presented in the supplemental material (S1 Table). A significantly higher number of bacterial species (observed diversity) was recorded in saliva samples (n = 177) than in site-specific (n = 134) and pooled subgingival samples (n = 132) (p<0.0001), whereas no significant differences in α-diversity (Shannon-index) was observed (p>0.05).

### Subgingival bacterial profiles display major intraindividual site-specific differences

**Fig 1:**
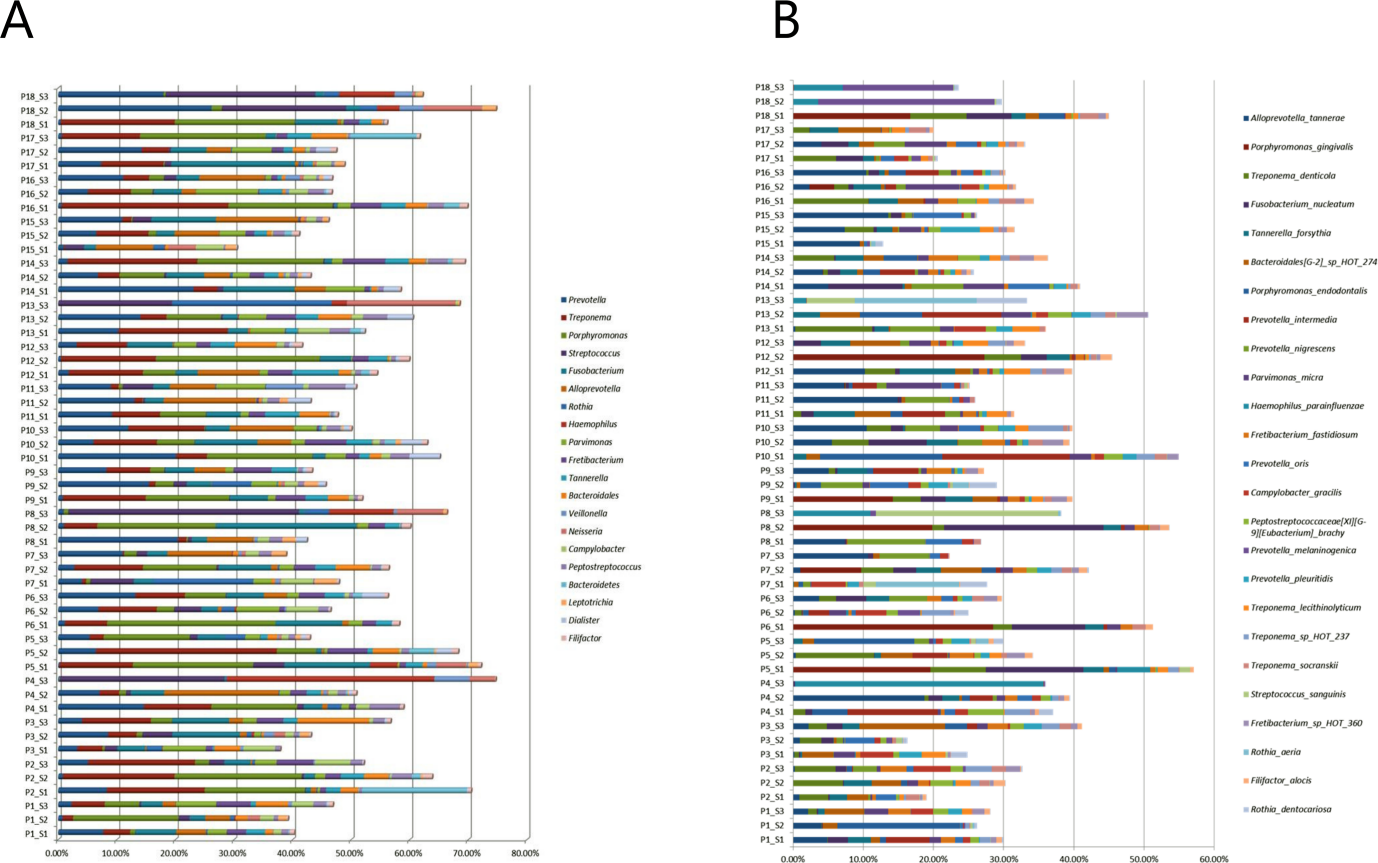
Site-specific subgingival microbiota. A: Relative abundance of the 20 predominant bacterial genera. B: Relative abundance of the 25 predominant bacterial species. Sample denotation: P1-P18: Person 1–18. S1-S3: Sample 1–3.

**Fig 2:**
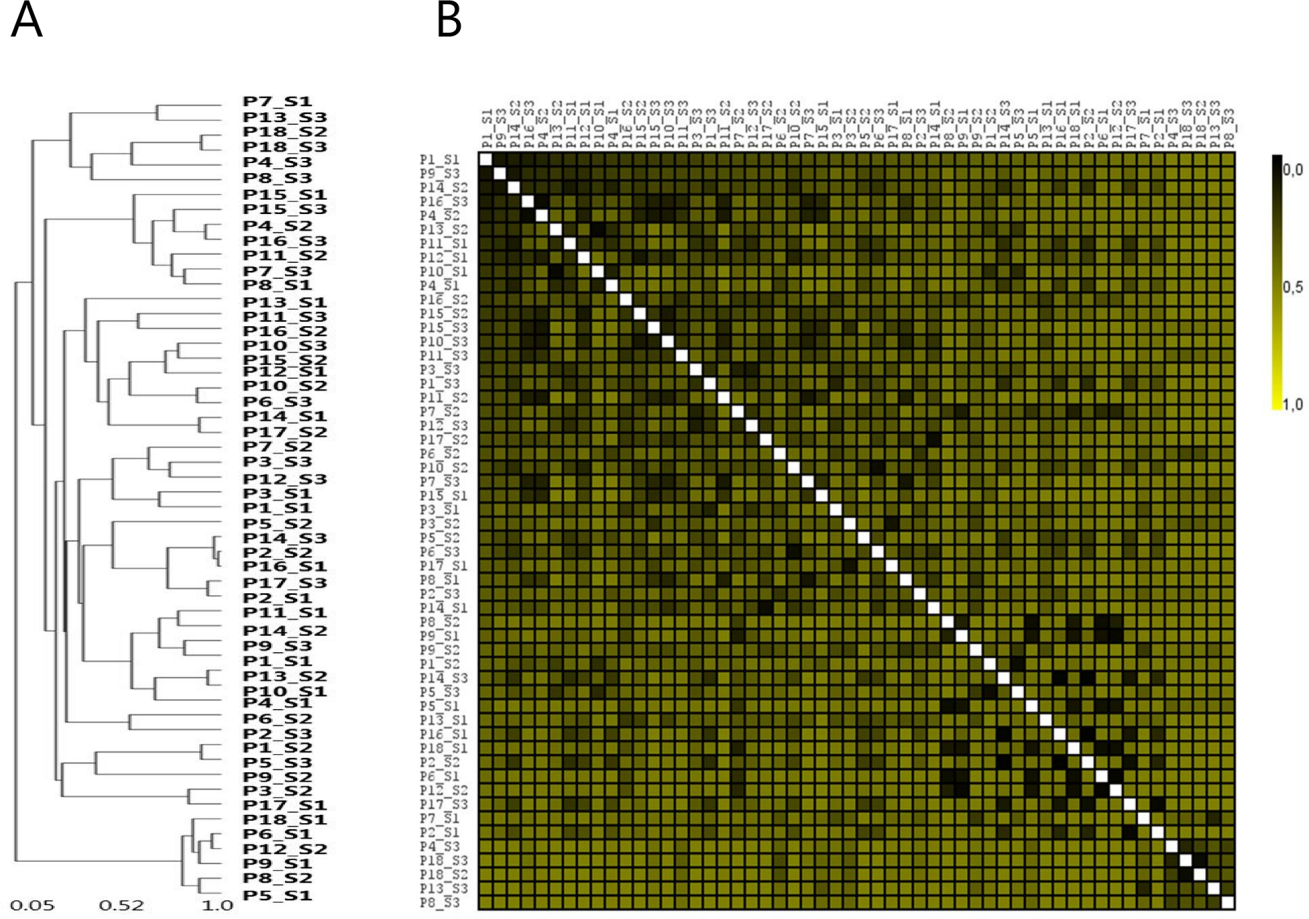
Clustering analysis of site-specific samples. A: Sample-tree clustering of site-specific samples. B: Heat-map of site-specific samples. Sample denotation: P1-P18: Person 1–18. S1-S3: Sample 1–3.

Relative abundance of the 20 predominant bacterial genera and 25 predominant bacterial species in site-specific subgingival samples is displayed in Fig 1A and 1B. The most predominant genera were *Prevotella*, *Treponema* and *Porphyromonas* constituting approx. 25% of the DNA reads with a high degree of intra- and interindividual variation. Comparison of correlation in microbial community profiles using Spearman rank correlation coefficient showed completely random distribution of site-specific subgingival plaque samples, with no tendency of clustering of samples collected from the same patient (Fig 2A and 2B).

### Pooled subgingival samples is not an average of site-specific samples

**Fig 3:**
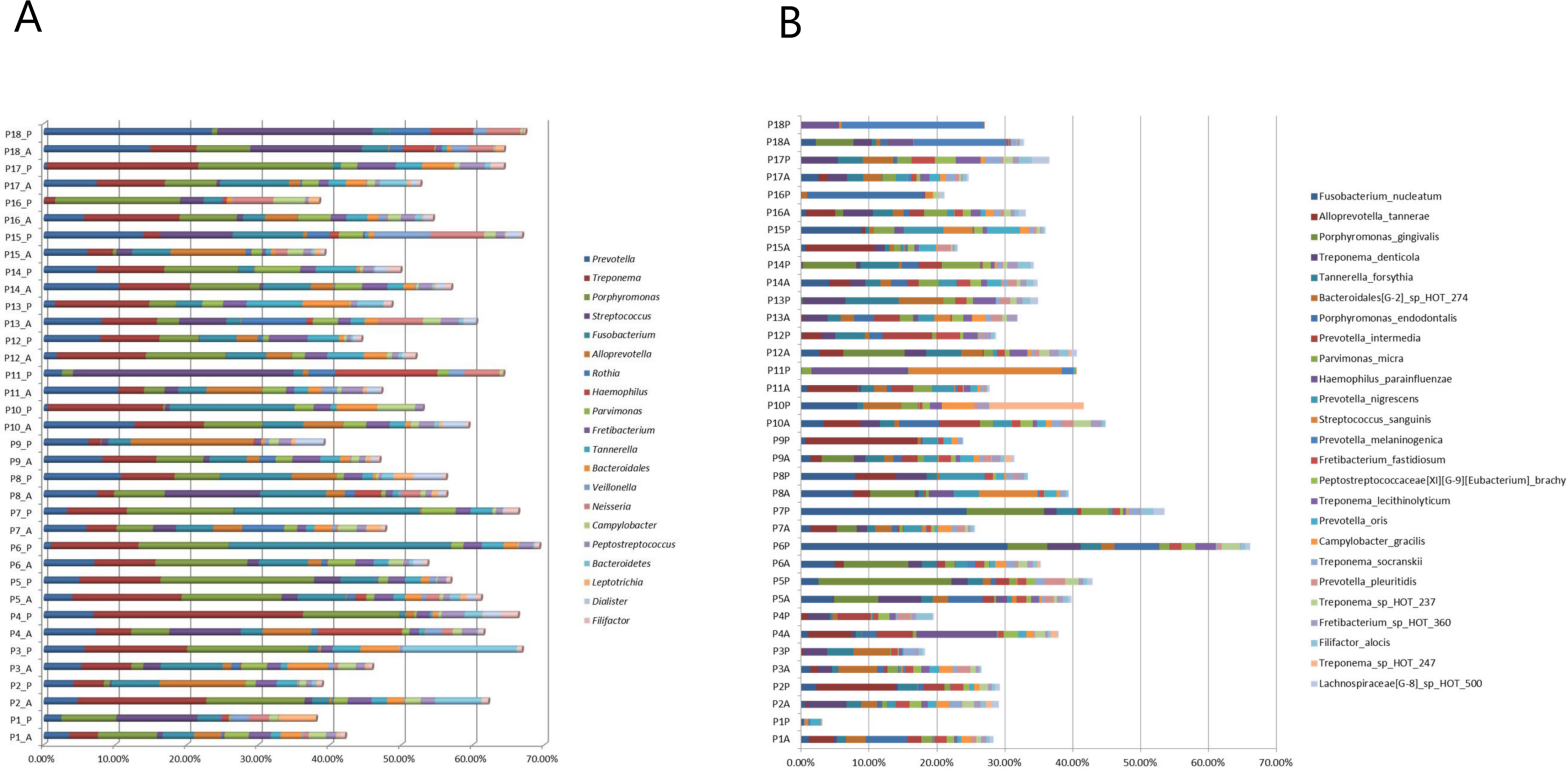
Pooled subgingival microbiota vs. average of site-specific subgingival microbiotas. A: Relative abundance of the 20 predominant bacterial genera. B: Relative abundance of the 25 predominant bacterial species. Sample denotation: P1-P18: Person 1–18. A: Averaged samples. P: pooled samples.

**Fig 4:**
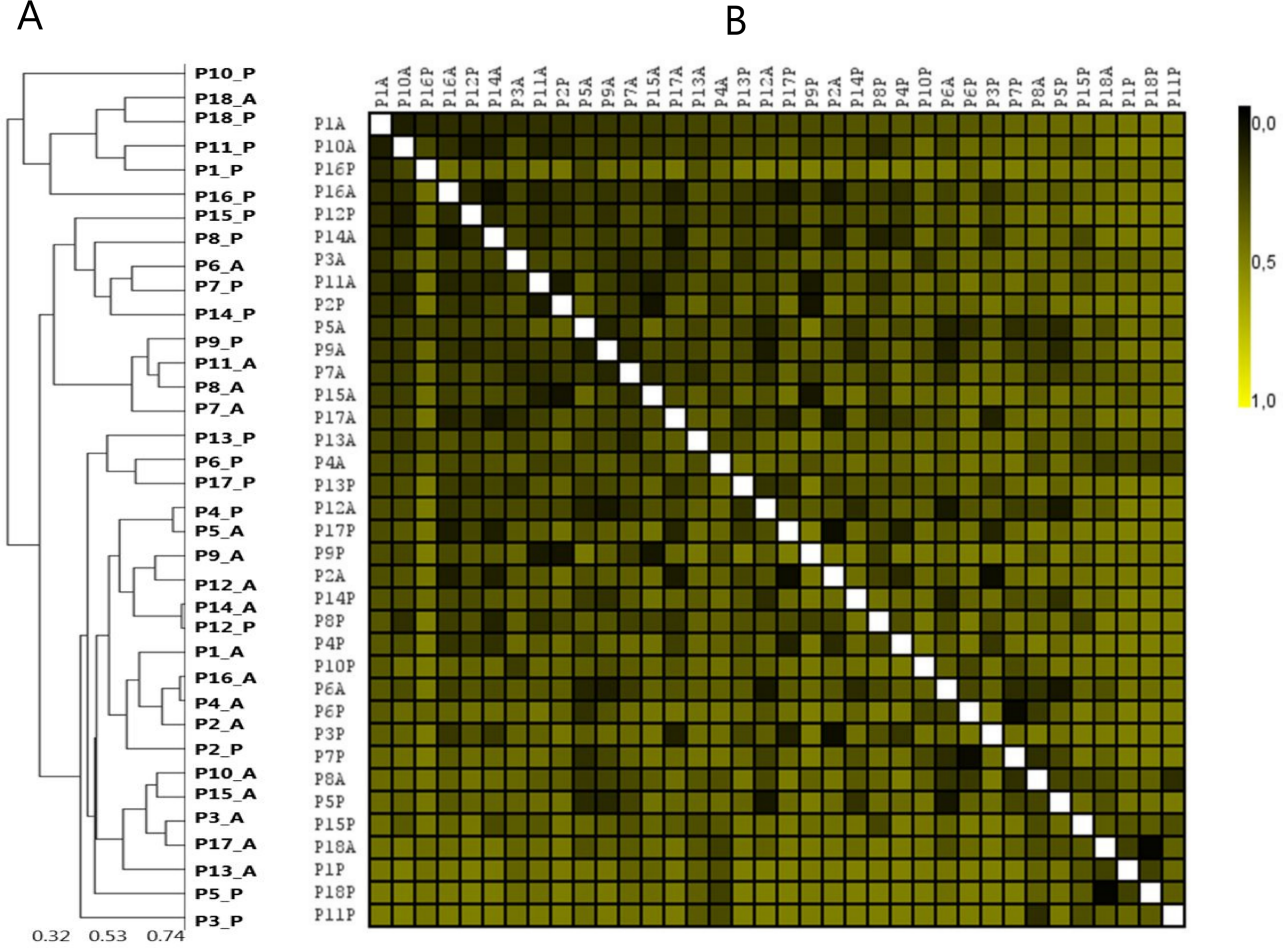
Clustering analysis of pooled and averaged samples. A: Sample-tree clustering of pooled and averaged site-specific samples. B: Heat-map of pooled and averaged site-specific samples. Sample denotation: P1-P18: Person 1–18. A: Averaged samples. P: pooled samples.

Comparable mean levels of the 20 predominant bacterial genera and 25 predominant bacterial species were identified in averaged site-specific samples and pooled subgingival samples (S1A and S1B Fig). However, major intra- and interindividual differences in the relative abundance of the 20 predominant bacterial genera and 25 predominant bacterial species were observed in averaged site-specific samples and pooled subgingival samples (Fig 3A and 3B). Spearman rank correlation coefficient displayed no correlation between averaged site-specific subgingival plaque samples and pooled subgingival plaque samples collected from the same patient (Fig 4A and 4B).

### Saliva is as proficient as pooled subgingival plaque samples to screen for periopathogens

Major differences in mean levels of the 20 predominant bacterial genera and 25 predominant bacterial species were observed in saliva compared to pooled subgingival samples and averaged site-specific samples (S1A and S1B Fig). Presence of *P*. *gingivalis*, *T*. *denticola*, *P*. *intermedia*, *F*. *alocis*, *T*. *forsythia* and *P*. *micra* in site-specific subgingival samples were compared to carriage of the same species in saliva and pooled subgingival samples collected from the same patient. In saliva samples, the 6 species were detected with an AUC of 0.79 (sensitivity: 0.61, specificity: 0.94), compared to an AUC of 0.76 (sensitivity: 0.56, specificity: 0.94) in pooled subgingival samples.

## Discussion

The purpose of the present investigation was to compare microbial community profiles of saliva, pooled and site-specific subgingival samples collected from the same patient. We tested whether saliva can be used as a surrogate for pooled plaque when screening for presence of periopathogens.

Subgingival colonization by specific bacterial species including *P*. *gingivalis*, *T*. *forsythia* and *T*. *denticola* are associated with periodontitis [6;8;29], and reduction of these specific bacterial taxa correlates with success of periodontal treatment [30]. Furthermore, the composition of subgingival biofilm in healthy sites in patients with periodontitis has been reported to differentiate from sites in healthy controls [12;31]. However, site-specific variation in subgingival profiles may be evident, as demonstrated by data from the present study (Figs 1A, 1B, 2A and 2B). These results were probably influenced by individual differences in pocket depth [32] and potentially by site-specific impact of smoking status [12]. Nevertheless, this finding illuminates individual diversity of subgingival microbiotas, and highlights the necessity for separate sampling and analysis of all healthy and diseased sites, when conducting studies aiming at characterization of the subgingival microbiota under various health status conditions. Obviously, translation of this setup in the routine clinical setting is hampered by the expensive and time-consuming nature of this approach.

Thus, pooling of subgingival samples has often been used to minimize the cost of microbial analysis in clinical periodontology. Pooled subgingival samples have been reported to sufficiently retrieve high numbers of bacterial counts, when compared to single-site subgingival plaque samples [33]. However, a pooled sample should include information from each site, and ideally be an average of data from each site combined. To test if this was the case, we mathematically computed an average sample based on combined information of relative abundance in site-specific samples. Data showed that in the majority of cases, a pooled sample was not an average of the samples combined, as pronounced differences in relative abundance of predominant genera and species in averaged and pooled samples were evident (Fig 3A and 3B). Thus, while pooled subgingival samples may be sufficient in clinical screening of bacterial resistance prior to antibiotic treatment, precaution should be taken when interpreting correlations of species specific data.

We used paper points to sample subgingival sites, as this method is routinely used for microbial analysis at Copenhagen University School of Dentistry. Furthermore, this sampling method has been reported to be as sufficient as curette sampling for microbial analysis of the subgingival microbiota [34]. Comparable numbers of sequences generated (pooled samples n = 43,952 vs. single-site samples n = 40,783, p>0.05), number of species identified (pooled samples n = 132 vs. single-site samples n = 134, p = 0.74) and α-diversity (pooled samples: 2.45 vs. single-site samples: 2.45, p = 0.88) was recorded in single-site and pooled subgingival samples, which suggests that the sampling strategy had a minimal impact on data.

In contrast to subgingival samples, saliva can easily be sampled with a high degree of reproducibility [22;35]. Salivary carriage of periodontal pathogens [16;36], and correlation of periopathogens in pooled subgingival samples and saliva samples has be reported in periodontitis patients [18–21]. Thus, even though the composition of the core salivary microbiota is different from that of subgingival plaque (S1A and S1B Fig)[4], salivary screening of specific periopathogens may be considered as a parameter in periodontitis risk assessment. However, a comparison on the efficacy of using saliva versus pooled subgingival samples for screening of site-specific presence of periopathogens has not been performed. Thus, we referenced identification of 6 periopathogens (*P*. *gingivalis*, *T*. *denticola*, *T*. *forsythia*, *P*. *intermedia*, *P*. *micra* and *F*. *alocis*) in saliva samples and pooled subgingival samples to that of single-site subgingival samples in each patient. Periopathogens were not identified in saliva if they were not simultaneously present in at least one site-specific subgingival sample. Furthermore, the 6 species were detected in saliva samples with an AUC of 0.79 (sensitivity: 0.61, specificity: 0.94) compared to single-site identification, which was comparable to an AUC of 0.76 (sensitivity: 0.56, specificity: 0.94) in pooled subgingival samples. Therefore, data from this study indicate that saliva may be a useful alternative to pooled subgingival samples in screening studies.

In conclusion, findings from this study underline the importance of always choosing a sampling strategy which strictly complies with the aim of the microbiological trial. Furthermore, saliva may be a reasonable surrogate for pooled plaque when screening for presence of specific periopathogens. Future large-scale studies are needed to confirm findings from this study.

## Supporting information

S1 Table List of identified bacterial genera and species. (XLSX)

S1 Fig Mean levels of bacterial genera and species. A: Mean relative abundance of the 20 predominant bacterial genera. B: Mean relative abundance of the 25 predominant bacterial species in pooled subgingival samples, averaged site-specific samples and saliva.
(PNG)
