# Supplementary figures and images for: Microbial profile comparisons of saliva, pooled and site-specific subgingival samples in periodontitis patients
Source: PLoS One. 2017 Aug 11;12(8):e0182992. doi: 10.1371/journal.pone.0182992 (PMC5553731; doi:10.1371/journal.pone.0182992)

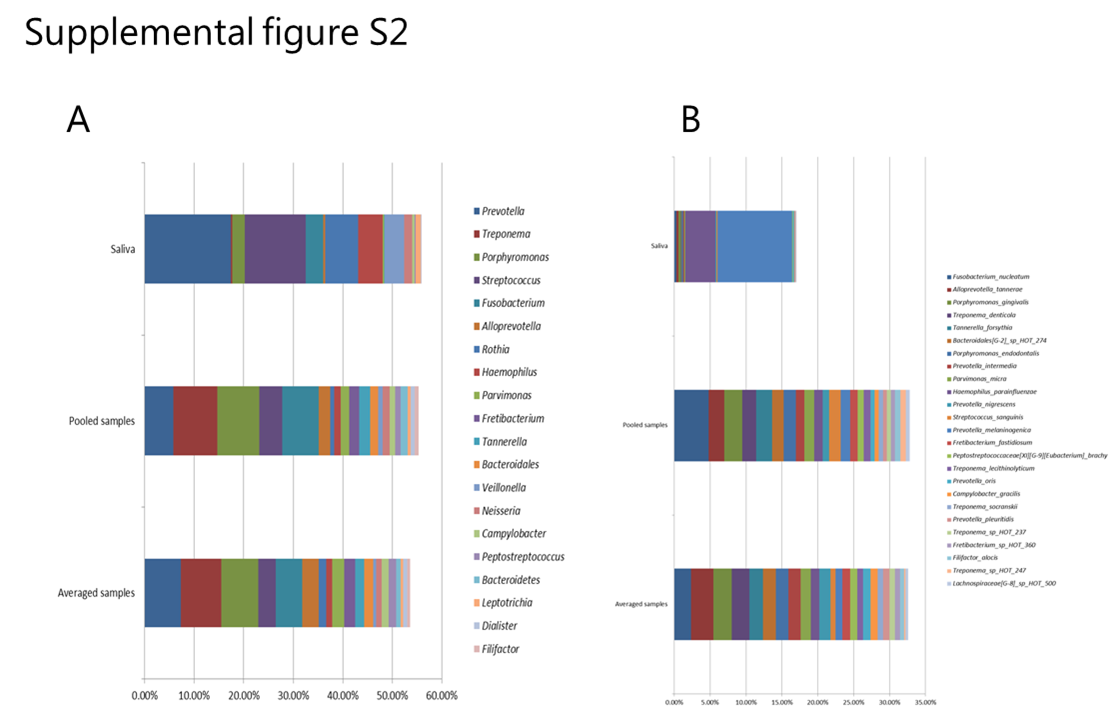

Supplement: S1 Fig — A: Mean relative abundance of the 20 predominant bacterial genera. B: Mean relative abundance of the 25 predominant bacterial species in pooled subgingival samples, averaged site-specific samples and saliva. (PNG) [file pone.0182992.s002.png]
